# HIV-1 restriction by SERINC5

**DOI:** 10.1007/s00430-022-00732-x

**Published:** 2022-03-25

**Authors:** Lucía Cano-Ortiz, Tom Luedde, Carsten Münk

**Affiliations:** 1 Clinic for Gastroenterology, Hepatology, and Infectiology, Medical Faculty, Heinrich-Heine-University Düsseldorf, Building 23.12.U1.82, Moorenstr. 5, 40225 Düsseldorf, Germany; 2 Laboratório de Virologia, Departamento de Microbiologia, Imunologia e Parasitologia, Instituto de Ciências Básicas da Saúde, Universidade Federal do Rio Grande do Sul, Porto Alegre, Rio Grande do Sul Brazil

**Keywords:** SERINC5, Nef, HIV-1, Restriction factors

## Abstract

Serine incorporator 5 (SERINC5 or SER5) is a multipass transmembrane protein with ill-defined cellular activities. SER5 was recently described as a human immunodeficiency virus 1 (HIV-1) restriction factor capable of inhibiting HIV-1 that does not express its accessory protein Nef (Δ Nef). SER5 incorporated into the viral membrane impairs the entry of HIV-1 by disrupting the fusion between the viral and the plasma membrane after envelope receptor interaction induced the first steps of the fusion process. The mechanisms of how SER5 prevents membrane fusion are not fully understood and viral envelope proteins were identified that escape the SER5-mediated restriction. Primate lentiviruses, such as HIV-1 and simian immunodeficiency viruses (SIVs), use their accessory protein Nef to downregulate SER5 from the plasma membrane by inducing an endocytic pathway. In addition to being directly antiviral, recent data suggest that SER5 is an important adapter protein in innate signaling pathways leading to the induction of inflammatory cytokines. This review discusses the current knowledge about HIV-1 restriction by SER5.

## Discovery of the SERINC5 anti-HIV-1 activity and Nef counteraction

Some host cellular proteins act as the first line of defense against infection of human immunodeficiency virus 1 (HIV-1) and other viruses by blocking directly different cellular steps of the viral replication cycle [1]. These host proteins also called restriction factors are part of the cellular innate immune system [2]. Well-described inhibitory proteins of HIV-1 include APOBEC3 cytidine deaminases [3–6], Tetherin/BST2 [7–10], SAMHD1 [11–13], and TRIM5α [14, 15]. Most recently, MX2 [16–19], SER3/5 [20–22], IFITM3 [23–25], SLNF11 [26] and MARCH2/8 [27, 28] have been reported. This review discusses the current knowledge about HIV-1 restriction by SER5. Studies focused on Nef function allowed the discovery of SER3 and 5 (SER3, SER5) as antiviral factors. Initially, it was observed that Nef from HIV-1 [29] and the glycoGag from the murine leukemia virus (MLV) [30] increase the infectivity of HIV-1 particles due to an unknown function in the virus producer cell. To understand these observations, in 2015, two independent studies with different approaches were able to identify these new restriction factors that are antagonized by the Nef. These experiments were based on proteomic analyzes [21] and transcriptional profiles [20] of cells permissive or resistant to HIV-1 not expressing Nef (∆ Nef) that allowed the identification of SER3 and SER5 proteins incorporated in virions generated from viral DNAs not encoding functional Nef [20, 21]. Besides HIV-1 Nef and MLV glycoGag, the S2 protein of the equine infectious anemia virus (EIAV) and also the glycoGag of the feline leukemia virus (FeLV) have been shown to counteract SER5 [20, 21, 31–33].

## SER genes and evolution

The serine incorporator (SERINC or SER) proteins are a family of eukaryote multi-transmembrane proteins highly conserved among different species [34], with recent evidence increasingly supporting their role in intrinsic immunity against retroviruses [22]. *S. cerevisiae* and *D. melanogaster* have only one gene of this family named TMS1. *C. elegans, C. intestinalis,* and *C. savignyi* encode likely two homologous genes to SER/TMS1: Y57E12AL.1 and R11H6.2 in *C. elegans;* F6YGN3 and F7A8J9 *in C. intestinalis* and*,* H2YQ96 and H2Z2X8 in *C. savignyi* [35, 36]. In mammals, the SER protein family comprises five members (SER1-5). Previous topologies of the SER encoding genes have shown that the genes amplified by two duplication events, one before and one after the vertebrate-invertebrate split [37] which gave rise to two clusters of genes encoding SER1/2/3 and SER4/5 [22, 37]. In humans, there are five alternatively spliced isoforms of SER5, SER5-001, -004, -005, -008a, and -008b, which differ in the region coding for the C-terminal end and the transmembrane domains [38]. SER5 (here we discuss isoform 001) is found in the plasma membrane and is composed of 10 transmembrane domains, five extracellular loops, and four intracellular loops (ICL) [39]. SER5-001 is the longest isoform (461 amino acids) and encodes for an additional transmembrane domain with an essential role in restricting HIV-1 replication [38]. In addition, human SER1/3/4 [20, 21, 40, 41], coelacanth SER2 [35], rodent and lagomorph SER3/5 [42], and feline SER5 [33] do have antiviral activity.

## Expression of SERINC proteins

In contrast to many other restriction factors, whose expression is regulated by interferons, it is interesting to note that the expression of SER5 is independent of interferon-induced signaling [20, 21, 43]. The immunodetection of SER proteins is still difficult due to the lack of specific antibodies to probe the endogenous level of these proteins [43]. SER5 is N-glycosylated at residue N294, which is critical for its steady-state level, and probably the non-glycosylated SER5 is degraded by proteasome as a quality control mechanism [44]. In mice, SER1 and SER3 are expressed in the central nervous system and SER3 mRNA is also found in the kidney and testis [45]. In humans, SER5 is ubiquitously expressed across tissues with a specific expression cluster in the liver (https://www.proteinatlas.org/ENSG00000164300-SERINC5/tissue), and the expression of SER5 is induced during the differentiation of monocytes to the myeloid lineage [43] as well as it is upregulated in oligodendrocytes during myelination [46]. In patients with HIV-1 compared to the uninfected individuals, the level of SER5 mRNA was found to be downregulated [47].

## Cellular functions of SERINC5

**Fig. 1:**
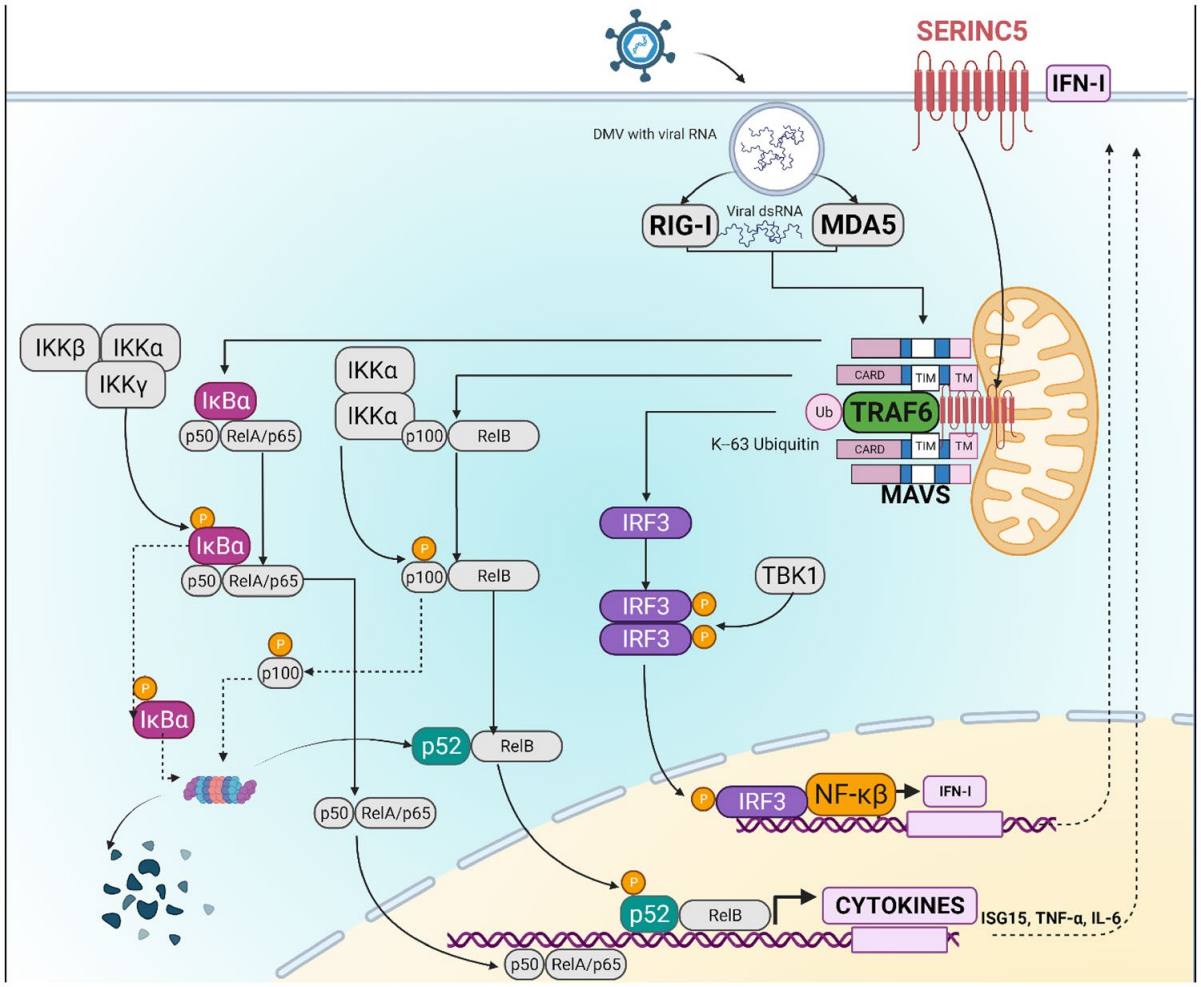
Model of IFN-1 and inflammatory pathways induced by SER5 according Zeng et al*.* [50]. After the viral infection by Sendai virus or the stimulation with poly (I:C) or LPS, SER5 relocates from the plasma membrane to the mitochondrial membrane. SER5 associates with the transmembrane domains of the mitochondrial membrane protein MAVS and with the E3 ubiquitin ligase TRAF6. TRAF6 associates also with MAVS. This stable complex is the result of the MAVS aggregation and the polyubiquitination of TRAF6. This complex could have three effects (1) the activation of the canonical NF-κB pathway: after the phosphorylation of the IκBα repressor, the complex p50/p56 (p65 also named RelA) is transported to the nucleus and induces the expression of pro-inflammatory genes like ISG15, TNF-α IL-6; (2) the activation of the non-canonical NF-κB pathway; and (3) the phosphorylation of IRF3 and the translocation of phosphorylated dimers to the nucleus to induce the activation expression of IFN-I

**Fig. 2:**
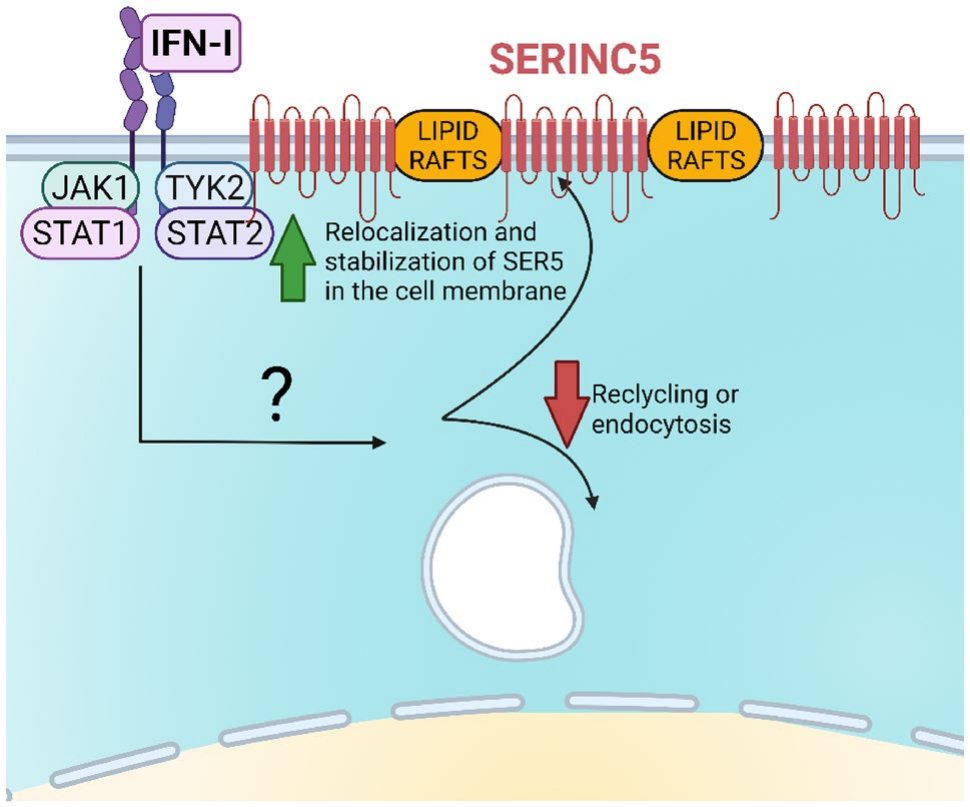
Model of the effect of IFN-I on SER5. Type I interferon increases the amount of SER5 in the plasma membrane by reducing its recycling or endocytosis. This effect can be antagonized by ruxolitinib, which inhibits the Jak/STAT signalization pathway. This IFN effect is not dependent on the modulation of mRNA or protein levels of SER5 [51]

Initially, SER proteins were characterized as carrier proteins, to incorporate serines into membrane lipids, such as phosphatidylserine and sphingolipids [48]. However, the knock-out of SER1 in some immune cells did not alter the serine lipid composition and function [49]. Moreover, SER5 did not alter the lipid composition in the HIV-1 particles [34]. Recently SER3 and SER5 were reported to have an intracellular role in innate sensing and signaling. Both SER3 and SER5 enhance the expression of type I IFN and the nuclear factor κB (NF- κB) contributing to the antiviral activity [50]. After infection with Sendai virus or stimulation with poly (I:C) or lipopolysaccharides (LPS), SER5 internalizes into the mitochondria membrane and associates with the mitochondrial membrane protein MAVS (mitochondrial antiviral signaling protein). As a result, MAVS aggregates and complexes with tumor necrosis factor receptor-associated factor 6 (TRAF6) through K63-mediated ubiquitylation. The complex MAVS-SER5-TRAF6 induces the phosphorylation of IRF3 and IκBα, which activates NF-κB (including the non-canonical pathway) and the expression of genes encoding type I IFNs and cytokines by activating the transcription factors interferon regulatory factor 3 (IRF3), IRF7, and NF-κB. Thus, SER5 functions here between MAVS and TBK1 [50] (Fig. 1). Interestingly, it was also reported that IFN-I stimulation enhances the surface levels of endogenous SER5 in a process that relocates the intracellular SER5 and stabilizes it on the plasma membrane. This was confirmed after observing the opposite effect by blocking the Jak/STAT signaling pathway with ruxolitinib [51] (Fig. 2). Those findings place SER5 as a factor involved in intracellular immune signaling pathways beyond its initially antiviral role associated with the plasma membrane.

## SERINC5 inhibits HIV-1

SER5 restricts HIV-1 replication when the virus does not express Nef (HIV-1 ∆Nef) [20, 21]. In contrast, human SER1 and SER2 display very limited or no activity against HIV-1 [40, 52]. However, SER2 from other species as coelacanth and *Xenopus* can inhibit HIV-1 [35]. These findings may indicate that the activity of SER2 was initially directed against retroviruses found in these species and it was lost during evolution. Further support for an ancient antiviral function of SER proteins comes from the finding that the envelope glycoprotein of the prototype foamy virus (a primate retrovirus that belongs to spumaviruses) counteracts the HIV-1 inhibition of coelacanth SER2 [35]. SER3 proteins from different species (human, primate, mouse, frog, and zebrafish) also moderately inhibit HIV-1 [21, 40, 42]. Despite the rapid turnover and low expression level of human SER4 (at least 250-fold lower than that of human SER5), this protein can also restrict HIV-1 as strongly as SER5 when the protein level is restored by transfecting 250-fold more SER4 than SER5 plasmids [40, 41].

In general, the antiviral activity of SERs is exerted on HIV-1 ∆ Nef viruses, only when SER proteins are packaged into viral particles [20, 21]. Various SER proteins have different abilities to incorporate into the budding virus membrane, where, SER5 is very efficiently associated in its glycosylated form [40, 44]. SER5 is localized in the detergent-resistant domains in the plasma membrane, where the budding of HIV takes place [40]. This indicates a passive mechanism of the SER5 association with the virus [22].

The restriction activity of SER occurs predominantly during the entry of the virus at the stage of fusion of viral and plasma membranes. Exactly how SER proteins block HIV infection of target cells is not fully understood, however, some hypotheses have been postulated [53–56]. It is possible that the infection of target cells is reduced by virion incorporated SER5 due to an alteration of the formation and/or enlargement of the fusion pore affecting the envelope-mediated fusion process [21, 57, 58]. In addition, it was suggested that SER5 proteins alter the conformation of the viral envelope and prevent the entry of the virus into the host cell [39, 52]. However, the fusion process between the cell and the HIV-1 WT or ∆ Nef has been found to have the same fusion capabilities independently of the SER5 expression status of the producer cells and thus, the inhibitory effect of SER5 may reduce further steps during the viral entry [51]. Regardless of Nef expression, several reports demonstrate that the antiviral activity of SER5 depends on an ill-defined sensitivity of viral Env proteins [58–60]. The HIV-1 trimeric envelope spike is formed by the Env surface subunit (gp120) with its five variable regions (V1 to V5) and the transmembrane subunit (gp41) with its cytoplasmic tail. The open conformation of the envelope protein seems to be essential for the SER5 restriction [59, 61] and SER5 appears to inactivate sensitive but not resistant Env glycoproteins [58, 59, 62, 63] that include some wildtype HIV-1 envelopes and variants with truncations of the cytoplasmic tail [64, 65].

Whether a direct interaction between SER and HIV-1 envelope is relevant is unclear [64]. SER5 could interact with Env in the plasma membrane and the extent of the binding correlates with the sensitivity of Env to SER5 [59]. It was suggested that Env-SER5 interaction is mediated by the extracellular loop 3 and 5 of SER5 which have a spacing that matches that between the MPER regions (membrane-proximal external region) within the HIV-1 Env trimer [39]. In contrast, it was also discussed that SER5 disrupts Env clusters without a direct binding and no co-distribution of Env-SER in virions was detected [66].

Envelopes from other viruses have been also demonstrated to be sensitive, such as amphotropic MLV and influenza A virus (IAV). In contrast, diverse viral surface glycoproteins *e.g.* derived from vesicular stomatitis virus (VSV-G), Ebola virus (EBOV), equine infectious anemia virus (EIAV), avian leukosis virus A (ALV-A), and feline leukemia virus subgroup B (FeLV-B) and the ecotropic MLV are resistant to the antiviral SER5 activity [31, 33, 65, 67]. In principle, the reason why some envelopes are sensitive to SER5 or insensitive is not clear [65]. Initially, it was postulated that envelopes with a low pH-dependent endocytic entry pathway were SER5 resistant [20, 21], however, this was not demonstrated with other glycoproteins [65]. In SER5 resistant HIV-1 envelopes, the resistance correlates to a closed conformation of Env where the variable loops V1–V3 interact with each other [52, 59, 66, 68]. Additionally, the viral core also seems to be an important factor that determines the sensitivity of some envelope proteins. For instance, the mason-pfizer monkey virus (M-PMV) glycoprotein is sensitive to SER5 when it is used to pseudotype MLV or M-PMV cores, but shows resistance when it is used on HIV-1 cores [65]. With other viral glycoproteins similar observations were made, where the SER5 resistance was viral core-dependent [65].

Another interesting antiviral function of SER5 that is also counteracted by Nef is an enhanced recognition of such HIV-1 particles by primary myeloid cells, which leads to the production of proinflammatory cytokines like granulocyte–macrophage colony-stimulating factor (GM-CSF), GRO alpha, IL-6, IL-8, and TNF-α. The mechanism of this virus recognition is not completely clear and may involve sensing of viral RNAs [69].

## Nef counteracts SERINC5

**Fig. 3:**
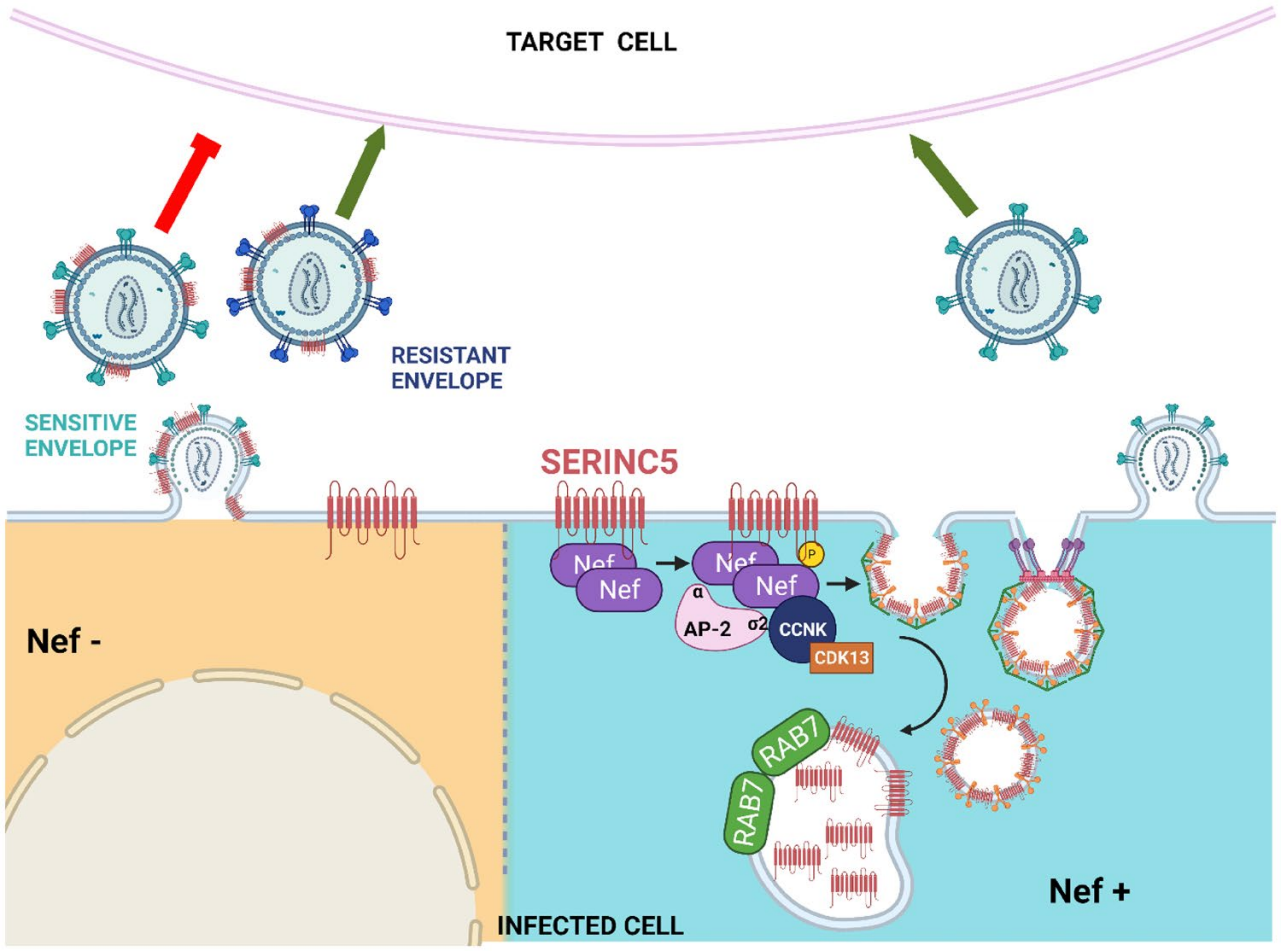
HIV-1 counteracts the antiviral activity of SER5 by its Nef protein.*.* When Nef is absent, the surface expression of SER5 in the plasma membrane is not altered and SER5 is incorporated into the newly formed viral particle. The infectivity of the virus will depend on its envelope. Virions with a SER5-sensitive envelope have an impaired infectivity while a resistant envelope allows the infection of the target cell even in the presence of SER5. If Nef is present, SER5 associates with the AP-2 complex and Nef with low affinity. Afterwards, Cyclin K/CDK13 is recruited and phosphorylates SER5 in the intracellular loop 4 (ICL4). In consequence, the AP-2 complex is recruited which induces the endogenization of SER5 to Rab7 + late endosomes, SER5 is downregulated from the plasma membrane and thus is not incorporated into the budding virion keeping its infectivity capacity [55]

HIV-1 Nef prevents the incorporation of SER5 into budding virions by inducing the down-regulation of SER5 from the plasma membrane [22]. Nef is an accessory protein of 27 to 32 kDa, encoded only by primate lentiviruses (HIV-1, HIV-2, and SIV) [70]. Nef was found to reduce the expression of several surface proteins in the infected cells, modulate signaling pathways in T cells and increase the infectivity of viral particles [71]. While most studies on ectopic expressed SER5 concluded that Nef is inducing cellular degradation of SER5, a study on the mRNA and protein levels of endogenous SER5 observed that Nef modulates only the SER5 surface localization without altering the steady-state levels of SER5 [51] (Fig. 3). Uniformly in all studies, HIV-1 Nef reduced the amount of SER5 on the cell surface by 70–90% [33, 51, 54, 72]. Myristoylated Nef associates with the plasma membrane as homodimers [54] where it interacts with SER5. In a second step, Cyclin K (CCNK) and cyclin-dependent-kinase 13 (CDK13) are recruited and the serine 360 in SER5 on the intracellular loop 4 (ICL4) is phosphorylated. This phosphorylation induces a conformational change in ICL4 that facilitates the interaction of SER5 with Nef by increasing the accessibility of residues L350/I352 to Nef which potentiates the binding [55]. When the conformational change is maintained, an unknown endocytic motif of ICL4 is exposed and the adaptor protein 2 (AP-2) is recruited [55]. The Nef C-terminal dileucine motif ExxxLL interacts with the AP-2 and the Nef N-terminal (amino acids 32–39) bind with SER5 [20, 21, 54, 73]. Consequently, Nef induces polyubiquitination of SER5 via a K48- and K63-linkage as a prerequisite for lysosomal degradation [74, 75]. This assembly, SER5, Nef, cyclin K/CDK13, and AP-2, causes endocytosis and lysosome-mediated degradation of SER5 from the membrane [55, 75, 76]. SER5 is relocated especially to Rab7 + late endosomes [20, 54, 74, 75], however, SER5 has been also found in Rab5 + early and Rab11 + recycling endosomes [75].

The activity of Nef proteins from the HIV-1 group M subtypes A, B, C, and D was found to be not identical and especially the subtype B Nef clones showed the highest function in SER5 internalization, followed by the Nef from subtype D, while Nef proteins of subtypes A and C displayed the lowest capacity to induce SER5 internalization [77]. The ability of Nef to counteract SER3 and SER5 varies among patients living with HIV-1. While most of the Nef proteins were capable of strongly antagonizing SER3 and SER5, some mutations were associated with a better counteraction of SER3 (N51T, H116N, and 188S) and SER5 (S163C) by increasing the HIV-1 infectivity [78]. The counteraction of SER5 by Nef was also associated with a lower viremia in HIV-1 infected patients [79]. Some natural polymorphisms in HIV-1 Nef from elite controllers (*e.g.,* K94E and H116N) were described to display lower infectivity and replication capacity in the presence of SER5 [80]. Other Nef polymorphisms, here in the dileucine motif, were found to be associated with the CD4 and SER5 downregulation. This finding supports a relation between Nef downregulation of SER5 and the rate of plasma CD4^+^ T cell decline [81]. However, a different study with the Nef of the simian immunodeficiency virus (SIV) showed that the activity against SER5 can be separated from the general downregulating functions of Nef. After mutating some important residues for counteracting SER5, Nef still reduced the amount of CD3, CD4, and MHC-I from the plasma membrane [82].

## Concluding remarks

In summary, the cellular functions of the SER proteins appear to keep some secrets, and solving these puzzles may be the solution to understand their antiviral activity. Different retroviruses evolved independent pathways to remove SER5 from the plasma membrane to prevent SER5 incorporation. HIV Nef, EIAV S2, and MLV/FeLV glycoGag are unrelated retroviral proteins that have in common that evolution independently directed them to SER5 counteraction. In addition, viral envelopes have been shown to have an important role in conferring resistance against SER5 inhibition. It is unclear why retroviruses use two strategies to escape SER5, but possibly suggesting a strong in vivo restriction by SER5 [67, 83].
